# Diabetic Ketoacidosis and Acute Mesenteric Ischemia in Adults: An Underreported Association

**DOI:** 10.7759/cureus.29053

**Published:** 2022-09-11

**Authors:** Gina R Vivino, Nicole A Crofton, Saad Mussarat

**Affiliations:** 1 Internal Medicine, Eastern Virginia Medical School, Norfolk, USA

**Keywords:** diabetes mellitus type 1, diabetes mellitus type 2, small bowel ischemia, sma thrombosis, metabolic acidosis, acute mesenteric ischemia, diabetic ketoacidosis (dka)

## Abstract

Acute mesenteric ischemia (AMI) is a rapidly fatal abdominal process that has been associated with diabetic ketoacidosis (DKA). This association has been reported among pediatric patients but has rarely been reported in adult patients. This case series presents two adult patients who presented with DKA and were subsequently found to have AMI. The first case is that of a 60-year-old male with a history of insulin-dependent type II diabetes mellitus who had a presentation and laboratory values consistent with DKA. He developed hypovolemic shock and worsening acidosis, and computed tomography angiography (CTA) revealed superior mesenteric artery occlusion. The second case is that of a 41-year-old male with a history of type I diabetes mellitus who presented with DKA and had no improvement despite aggressive resuscitation with fluids and insulin therapy. Computed tomography (CT) imaging of the abdomen revealed pneumatosis of the small bowel, indicative of ischemia. Both patients underwent bowel resection in the operating room. Given the high mortality and morbidity of AMI, it is an important diagnosis to consider in patients with DKA who have unresolving acidosis.

## Introduction

Diabetic ketoacidosis (DKA) is an acute, life-threatening complication of diabetes mellitus. Many cases of DKA occur among patients with type 1 diabetes; however, DKA in type 2 diabetes is also possible. DKA results from insulin deficiency and the body’s inability to use glucose for energy. Patients in DKA present with hyperglycemia, ketoacidosis, and ketonuria. Both diabetes and diabetic ketoacidosis promote hypercoagulable states [1,2]. The mortality of diabetic patients is largely due to cardiovascular disease, cerebrovascular accidents, and peripheral vascular disease [2]. Acute mesenteric ischemia (AMI) is a less common, but potentially fatal, complication of DKA. This association has been reported among pediatric patients [3,4]; however, it has been rarely reported in the adult population. We report a case series of two adult patients who presented with severe DKA and whose course was complicated by AMI.

## Case presentation

Case 1

This patient is a 60-year-old male with a history of insulin-dependent type II diabetes mellitus, hypertension, peripheral artery disease status post left above the knee amputation, and chronic obstructive pulmonary disease (COPD) who presented to the emergency department (ED) via emergency medical services (EMS) after a home health nurse found him in an altered mental status on the floor of his home. The patient was a poor historian but reported three days of non-bloody diarrhea, nausea, and vomiting.

In the ED, his vitals were notable for a heart rate of 126 beats per minute (60-100 bpm), blood pressure of 123/71 (<120/80), respiratory rate of 18 breaths per minute (12-20), and O_2_ saturation of 99% on room air (95-100). On exam, the patient had blood in his mouth and right nares with no other signs of active bleeding. He had coarse breath sounds throughout the lung fields on auscultation. His abdomen was soft, non-tender, and non-distended, and there was no rebound or guarding. He was confused but calm and intermittently followed commands with no focal neurological deficits.

Labs were significant for severe hyperglycemia with blood glucose levels greater than 1400 mg/dL, a high anion gap acidosis with a pH of 7.12, and an anion gap of 48.1 mmol/L. Additional pertinent labs are highlighted in Table 1. The patient was admitted to the intensive care unit (ICU) for the management of severe DKA.

**Table 1:** Blood results on admission for Case 1

Parameters	Admission	Reference Values
White cell count (x10^3^/uL)	18.9	4.0-11.0
Hemoglobin (g/dL)	12.6	13.1-17.2
Hematocrit (%)	40.9	39.3-51.6
Platelet (K/uL)	376	140-440
Segmented neutrophils (%)	92	40-75
Glucose (mg/dL)	1430	70-99
Urea nitrogen (mg/dL)	79	6-22
Creatinine (mg/dL)	3.2	0.5-1.2
Sodium (mmol/L)	121	133-145
Potassium (mmol/L)	5.6	3.5-5.5
Chloride (mmol/L)	66	98-110
Bicarbonate (mmol/L)	7	20-32
Anion gap (mmol/L)	48.1	3-15
Beta-hydroxybutyrate (mg/dL)	>140	0.2-2.8
Lactic acid (mmol/L)	6.4	0.5-2.0
pH	7.12	7.35-7.45

**Figure 1:**
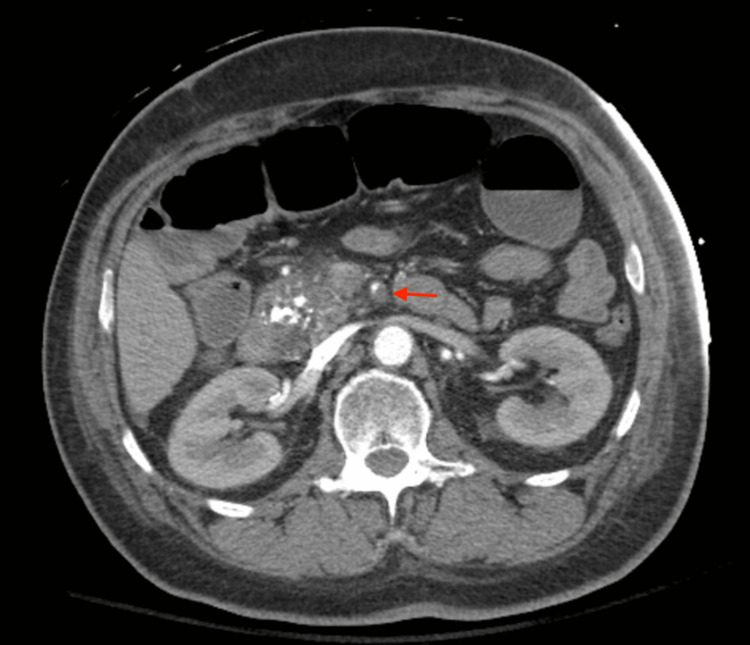
CTA showing a filling defect in the SMA (red arrow), suggestive of SMA occlusion CTA: Computed tomography angiography; SMA: Superior mesenteric artery.

On the evening of admission, his course was complicated by large-volume coffee-ground emesis followed by progressive hypothermia and acute respiratory failure that required emergency intubation. He was started on vasopressor support due to his shock. It was initially thought to be hypovolemic in the setting of diabetic ketoacidosis and emesis. However, he did not respond to aggressive fluid resuscitation and required increased pressor support. His labs showed worsening lactic acidosis even with improved hemodynamics. Computed tomography angiography (CTA) of the chest, abdomen, and pelvis was ordered to rule out bowel ischemia. His CTA showed superior mesenteric artery (SMA) occlusion and pneumatosis of the cecum and distal small bowel suggestive of mesenteric ischemia (Figure 1).

He was taken into surgery for emergent management consisting of exploratory laparotomy, subtotal colectomy, and retrograde open SMA stenting. The following day, the laparotomy was reopened, and ileostomy, abdominal wall closure, and Prevena™ vac placement were performed. He made a good recovery after his surgery. He was transitioned to a regular diet and discharged with outpatient general surgery follow-up.

Case 2

This patient is a 41-year-old male with a past medical history of type I diabetes mellitus, malnutrition, homelessness, hypertension, congestive heart failure, and chronic kidney disease who presented to the ED via EMS after a bystander found him unresponsive at a bus station. Per the report, he was responding only to painful stimuli. The patient had a history of multiple admissions for DKA secondary to medication noncompliance. The most recent had been one week prior to this presentation, during which he was also treated for *Clostridioides difficile* colitis.

On admission, his vitals were notable for a heart rate of 74 beats per minute (60-100 bpm), blood pressure of 111/61 (<120/80), respiratory rate of 28 breaths per minute (12-20), and O_2_ saturation of 100% on room air (95-100). On exam, the patient appeared disheveled, was cachectic, and was moaning in his bed. His mucus membranes were dry, and he had bilateral parotid gland swelling. He had deep, rapid, and labored breathing, and his lungs were clear to auscultation bilaterally. His abdomen was soft, non-tender, and non-distended, and there was no rebound or guarding. His eyes would spontaneously open, and he occasionally followed commands.

Labs were consistent with DKA. They showed severe hyperglycemia with blood glucose levels greater than 1,500 mg/dL, a high anion gap acidosis with a pH of 6.7, and an anion gap of 37 mmol/L. There was severe hyponatremia with a sodium of 108 mmol/L. Additional pertinent labs are highlighted in Table 2. The patient was admitted to the ICU for the management of DKA with severe metabolic derangements.

**Table 2:** Blood results on admission for Case 2

Parameters	Admission	Reference Values
White cell count (x10^3^/uL)	26.3	4.0-11.0
Hemoglobin (g/dL)	7.1	13.1-17.2
Hematocrit (%)	33.1	39.3-51.6
Platelet (K/uL)	501	140-440
Segmented neutrophils (%)	89	40-75
Glucose (mg/dL)	>1,500	70-99
Urea nitrogen (mg/dL)	45	6-22
Creatinine (mg/dL)	3.1	0.5-1.2
Sodium (mmol/L)	108	133-145
Potassium (mmol/L)	7.1	3.5-5.5
Chloride (mmol/L)	66	98-110
Bicarbonate (mmol/L)	4	20-32
Anion gap (mmol/L)	37	3-15
Beta-hydroxybutyrate (mg/dL)	89.1	0.2-2.8
Lactic acid (mmol/L)	9.5	0.5-2.0
pH	6.7	7.35-7.45

On the day following admission, the patient showed no progression in the treatment of his DKA despite aggressive fluid resuscitation and insulin therapy. His course was complicated by hypotension needing support with multiple vasopressors. He then developed acute respiratory distress requiring emergency intubation.

**Figure 2:**
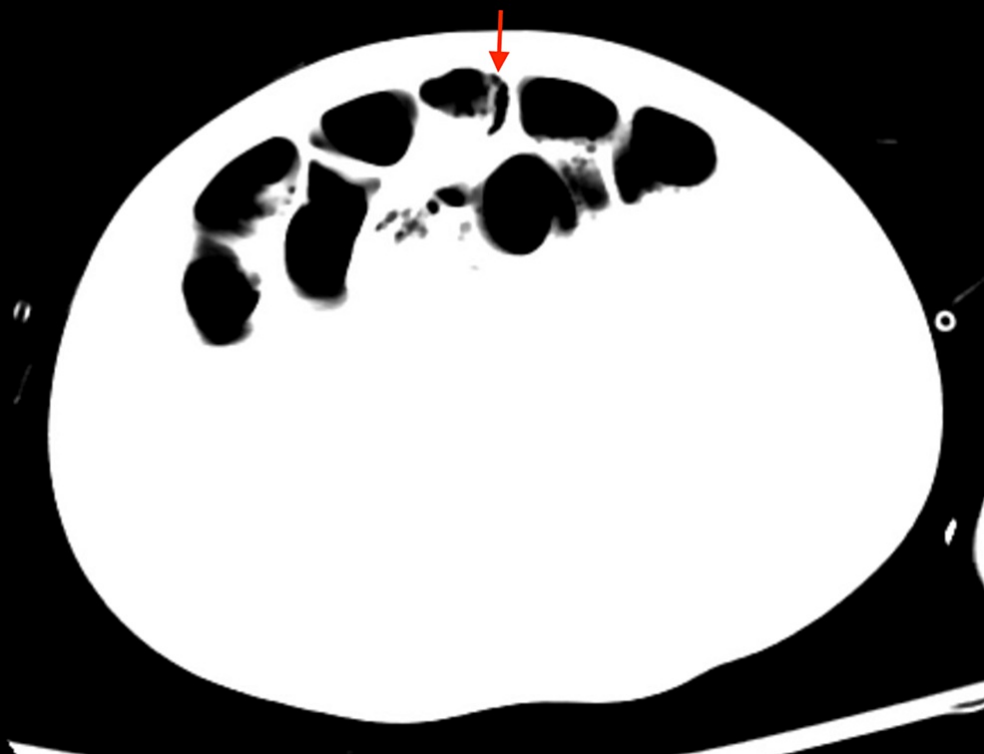
CT showing dilated small bowel with pneumatosis (red arrow), highly suggestive of severe ischemia CT: Computed tomography.

CT scan of the abdomen showed a significant amount of free fluid in the abdomen and multiple loops of small bowel with pneumatosis (Figure 2). He was taken to surgery for exploratory laparotomy and small bowel resection. Intra-operation findings demonstrated that most of his small bowel was cyanotic and necrotic, and he was left with approximately 110 cm of the bowel.

This patient had a prolonged post-surgical hospital course that was complicated by failure to thrive and a need for renal replacement therapy. Ultimately, he was discharged to a skilled nursing facility with the need for enteral feeding due to his malnutrition.

## Discussion

AMI is associated with diabetic ketoacidosis. However, many of the cases have been reported in pediatric patients. Our case series is unique because we highlight two adult patients who had similar presentations of DKA complicated by AMI. Nicol and Davis [5] described a case of a well-controlled, insulin-dependent diabetic woman who presented with acute onset abdominal pain, nausea, and vomiting and was found to be with DKA. Emergency exploratory laparotomy revealed ischemic bowel, but the patient subsequently underwent cardiac arrest and could not be resuscitated. Chan-Cua et al. [3] presented a case of an adolescent with insulin-dependent diabetes mellitus (IDDM) who developed distal ileal necrosis from non-occlusive mesenteric ischemia after the resolution of DKA. DiMeglio et al. [4] described an infant with non-occlusive mesenteric ischemia that occurred from hypovolemia in the setting of DKA. Interestingly, painless mesenteric infarction secondary to SMA occlusion has been seen in an adult patient with diabetic ketoacidosis who had symptoms of paralytic ileus [6].

Diabetes mellitus has been well-researched as a hypercoagulable state. Patients with diabetes have been shown to have increased platelet aggregation, increased clotting factors, and decreased levels of anticoagulant protein C [2]. Diabetic ketoacidosis has been shown to promote a pro-thrombotic state via elevations in von Willebrand factor and decreased protein C and protein S concentrations [1]. The clot-activating factors in DKA, along with the abnormal vascular endothelium seen in diabetes, are associated with an increased risk of thrombotic events.

AMI is caused by embolic occlusion (40%-50%) or thrombotic occlusion of a previously stenotic vessel (20%-35%) or associated with dissection or inflammation of a mesenteric artery (5%) [7]. Some cases of AMI can be due to venous thrombosis. Non-occlusive mesenteric ischemia (5%-15%) is related to low volume states and decreased perfusion of the mesentery [7]. Mesenteric ischemia can also be chronic, resulting from atherosclerotic disease that progresses over time. Both thrombotic and non-occlusive AMI secondary to DKA have been demonstrated [3,5]. In our case series, both patients developed hemodynamic instability requiring vasopressors prior to exploratory laparotomy. In our first case, SMA occlusion was seen on imaging. Therefore, both non-occlusive and thrombotic causes of mesenteric ischemia are important to consider.

As seen in the literature, patients with DKA may initially present with ischemic bowel or it may develop after beginning the treatment of DKA. AMI often presents with “pain out of proportion to examination” and tenderness to abdominal palpation, which may be hard to distinguish in the setting of DKA where abdominal pain, nausea, and vomiting are common. If fluid resuscitation and resolution of acidosis in DKA do not improve abdominal symptoms, then it is important to consider ischemic bowel because it is a life-threatening diagnosis with high morbidity and mortality. Additionally, clinicians should have a high index of suspicion for AMI in patients with DKA who do not improve with treatment, even in the absence of abdominal symptoms. CTA is a first-line imaging choice because it is fast, noninvasive, and highly accurate [8]. Treatment is aimed at re-vascularization of the ischemic tissue and resecting the necrotic bowel [7]. Thus, clinicians should have a low threshold to perform a CTA abdomen and pelvis for early diagnosis as it can make a huge difference in bowel salvage.

In both cases presented in this article, severe metabolic acidosis did not improve despite initial efforts with insulin and fluid resuscitation. Further workup revealed ischemic bowel in both patients. AMI has a mortality rate of 60%-80% [7]. Patients often have complications after bowel resection, such as ileus, diarrhea, and malabsorption. Short gut syndrome and failure to thrive were complications seen in our second case. Some patients require long-term dietary needs with total parenteral nutrition. Given the morbidity and mortality associated with mesenteric ischemia, early identification and treatment are critical to improving outcomes.

## Conclusions

In this case series, we describe two adult patients with unresolving DKA who were subsequently found to have AMI. DKA itself is a life-threatening complication of diabetes mellitus. AMI should be considered in patients with DKA who have unresolving acidosis and/or unresolving abdominal pain. Early identification of mesenteric ischemia with CT angiography is critical to improving outcomes.

## References

[1] Carl GF, Hoffman WH, Passmore GG, Truemper EJ, Lightsey AL, Cornwell PE, Jonah MH (2003). Diabetic ketoacidosis promotes a prothrombotic state. Endocr Res.

[2] Carr ME (2001). Diabetes mellitus: a hypercoagulable state. J Diabetes Complications.

[3] Chan-Cua S, Jones KL, Lynch FP, Freidenberg GR (1992). Necrosis of the ileum in a diabetic adolescent. J Pediatr Surg.

[4] DiMeglio LA, Chaet MS, Quigley CA, Grosfeld JL (2003). Massive ischemic intestinal necrosis at the onset of diabetes mellitus with ketoacidosis in a three-year-old girl. J Pediatr Surg.

[5] Nicol KK, Davis GJ (1997). An unusual complication of diabetes mellitus: the zebra that became a horse. South Med J.

[6] Selby CD, Dennis MJ, Whincup PH (1987). Painless mesenteric infarction in patient with diabetes mellitus. Diabetes Care.

[7] Clair DG, Beach JM (2016). Mesenteric ischemia. N Engl J Med.

[8] Oliva IB, Davarpanah AH, Rybicki FJ (2013). ACR appropriateness criteria® imaging of mesenteric ischemia. Abdom Imaging.

